# Looking through the lens of reproductive justice: the need for a paradigm shift in sexual and reproductive health and rights research in Canada

**DOI:** 10.1186/s12978-021-01169-w

**Published:** 2021-06-23

**Authors:** Dina Idriss-Wheeler, Ieman M. El-Mowafi, Karine Coen-Sanchez, Abdiasis Yalahow, Sanni Yaya

**Affiliations:** 1 Interdisciplinary School of Health Sciences, University of Ottawa, Ottawa, Canada; 2 NorImpact, Stavanger, Norway; 3 School of Social Sciences, University of Ottawa, Ottawa, Canada; 4 School of International Development and Global Studies, University of Ottawa, Ottawa, Canada; 5 The George Institute for Global Health, Imperial College London, London, UK; 6 School of International Development and Global Studies, Faculty of Social Sciences, University of Ottawa, 120 University Private, Ottawa, ON K1N 6N5 Canada

In order to begin understanding the current context, producing culturally meaningful findings, and creating equitable health outcomes in the sphere of sexual and reproductive health and rights (SRHR) in Canada, we must first consider how, with whom and by whom research is conducted. As part of a series of commentaries on Reproductive Justice in Canada in *BMC Reproductive Health*, in this segment, we echo the Research Working Group of the Black Mamas Matter Alliance (BMMA) in emphasizing that *SRHR research* related to Indigenous, Black and People of Colour (IBPOC) must be rooted in a Reproductive Justice framework that includes social justice and human rights [1]. We call for IBPOC community voices to be heard, information to be accessible, narratives to be representative, and communities to have “control over production, documentation, possession and dissemination of their own data or stories” [2]. The authors of this commentary argue that a paradigm shift is needed in SRHR research, one that considers how Canadian institutions conduct, fund, and disseminate research on Indigenous, Black and racialized populations in Canada and internationally.

## Systemic racism within academic structures: barriers to entry and retention of Indigenous, Black and racialized students and researchers

Before systemic racism can be addressed, it must first be acknowledged. The culture around racism in Canada embraces a stance of colour-blindness, which simultaneously gaslights racialized people and excuses Canadians from addressing their colonial history. Indeed, the Canadian government has only recently found the political will to admit to the systematic oppression of Indigenous populations, and nearly half of Canadians surveyed believe that anti-Black racism is “no longer a problem” [3]. Therefore, it is not surprising that students and faculty continue to witness Canadian post-secondary institutions release hastily constructed, platitudinous statements about diversity and inclusivity in response to the current civil rights movement, with little to no meaningful action or accountability, thereby maintaining systemic racism. As sites of research and progressive thought, institutions of higher learning and academia should be at the forefront of dismantling systems of racism; instead, they continue to sustain and perpetuate white privilege.

Even before entering academic and research institutions, historical policies of oppression have led to Indigenous, Black and People of Color (IBPOC) students experiencing intergenerational disparities in accumulated wealth, knowledge, and networks compared to their white counterparts [3]. Indeed, Black and Indigenous students are noticeably performing worse in secondary education due to assessment bias from teachers, disproportionate suspensions and expulsions, a lack of diverse staff as role models, and little trust or feeling of belonging [3]. These inequalities follow them into their university careers as students and subsequently as researchers, with universities advertising a false narrative of equal opportunity in the academic sphere. In reality, IBPOC students, faculty, and staff experience barriers analogous to those in secondary education. Additionally, racialized students endure overt discrimination and racism that are hastily minimized or outright dismissed, affecting their educational experience and negatively impacting their academic and personal success [4].

Henry and Tator (2009) state that racialized faculty and students are denied equity and access through “the everyday values and norms, discourses, and practices within a dominant white Anglocentric, Eurocentric and racist culture” [5]. This is evidenced through the lack of racialized faculty representation at the post-secondary level in Canada. In their 2018 report, the Canadian Association of University Teachers (CAUT) indicated that 21% of university teachers are racialized, of whom 2% are Black and 1.4% are Indigenous [6]. Even then, racialized faculty are disproportionately hired on a part-time basis, further limiting their power, influence, and job security in academic institutions. As a result, racialized professors are less able to access the privileges of tenure—as their white colleagues do—and are therefore less able to advocate for their own safety and security [7]. They name structural barriers, discriminatory practices, preference for sameness, as well as unacknowledged and unconscious biases as culprits that negatively impact the career trajectories and legitimate participation of racialized and Indigenous scholars—particularly womxn of colour—in academia [8].

Furthermore, racialized part-time professors seeking a career in academia are often overburdened due to the multitude of unpaid labour, a high demand for mentorship of IBPOC students, and “tokenistic” representation on committees. Consequently, racialized professors are limited in their ability to dedicate time to their research, publishing, and securing funding—all of which are critical components of the current matrices used to assess applicants for tenure track positions. As Henry et al. (2016) demonstrated, equity policies in Canadian institutions of higher education are failing to create the diverse faculty and student body reflective of the broader multicultural Canadian milieu [5]. In their current form, academic institutions are inherently racist structures that silence IBPOC academic voices. They require fundamental dismantling and restructuring, lest these issues continue to be perpetuated for generations, thereby maintaining and solidifying white privilege in education and research.

## How can Canada’s health research funding system do better for Indigenous, Black and racialized faculty and students?

Datta et al. (2021) identify racism as a predictor of health inequities and call for a transformation of race-based health research in Canada [9]. The authors argue that actionable changes are necessary and should include recognition by the Canadian health services research community that racism, not race, is a social determinant of health. Concurrently, funding, government and philanthropic entities should invest in racialized communities, and provide IBPOC students, faculty and researchers with the necessary resources to enable them to affect change in their communities [2]. As Canadian Institutes of Health Research (CIHR) begins to engage in consultations regarding systemic racism in Canada’s health research funding system, it is fundamental that transparency and accountability measures are set in place. Institutions have been providing lip-service to IBPOC scholars about addressing racism and changing structures that perpetuate racism—yet it persists. Outcomes from these consultations must be radical, tangible, and implementable—anything else is unacceptable. Additionally, CIHR needs to model fair and equitable behaviour in their consultation process by treating IBPOC students, faculty, and researchers like any other scientific expert, and compensate them for their time and labour.

In 2018, CIHR, among other Tri-Council agencies, pledged to collect race-based data on successful recipients of grants among researchers and students; this disaggregated data has yet to be released [10]. The authors suspect that the data will show disparities in grant awards by race. Furthermore, as gender disparities have been shown in grant peer review processes as well as grant and personal award success rates [11], we predict that Black or Indigenous womxn—who make up a fraction of female health services researchers—have even less success than their white counterparts.

## Disaggregated data: what is the story in Canada?

One of the most frustrating and debilitating obstacles for researchers, advocates and policymakers in Canada working toward health equity, and SRHR more specifically, is the lack of rigorous quantitative disaggregated data.^1^ The importance of disaggregated data has been recognized for at least a century—W.E.B. Du Bois remarked that investing in disaggregated data is a necessary tool to combat economic and health inequities that result from racism and racial inequities [12].

Government agencies as well as research and policy entities in several countries—examples include US, UK, Finland—have managed to mandate race-based data collection [13], but Canada does not have a national policy to do so. There is, however, local, regional and provincial variation in race-based data collection. The City of Toronto, Ontario, Manitoba and Quebec are mobilizing their own race-based data collection in light of the COVID-19 pandemic to provide evidence-based decisions regarding interventions and programs that prioritize or target marginalized populations during the pandemic [14]. Furthermore, the Canadian Institute for Health Information (CIHI) has recognized the issue and continues to work on developing standards for race-based and indigenous identity data collection and reporting [15]. As such, Canadian researchers and advocates are forced to use non-standardized, non-comparable data about certain regions or US data as analogues for population level decisions in the Canadian context. This may be a starting point, but relevant decisions, policies, interventions and programs will be successful only when based on the unique needs of the racialized populations across communities in Canada. The lack of disaggregated data reinforces the culture of colour-blindness. We continuously shift focus towards the US for its racial inequities but fail to recognize that criticism is only possible because the US has disaggregated data to critique. Canada’s lack of disaggregated data is not evidence of a post-racial society, rather it is the definition of structural racism.

Disaggregating data for Indigenous populations by First Nations (with and without Indian^2^ “status”), Métis and Inuit communities is paramount in the quest for SRHR equity. The ability of Indigenous people to conduct research in their own communities is contingent upon the ability to self-identify as a member of that community. Indigenous people need to have determination over their own identities, rather than being granted that identity by the Canadian government as per the Indian Act—a carryover piece of legislation imposed by colonists to control the movement, language, resources, and culture of Indigenous persons. This being said, we must also be cognizant of *race-shifting* and *self-Indigenizing settlers*, a new and challenging social phenomenon that describes “white, French-descendant people discovering an Indigenous ancestor born over 300 years ago” with no connection or accountability to Indigenous Nations and communities laying claim to unceded territory and Indigenous rights [16, 17]. The Yeollowhead Institute—a First Nations-led research centre at Ryerson University—provides a protocol for working with Indigenous Communities in the academic setting. Components and commitments to relationship building are outlined as a beginning to developing “culturally appropriate, reciprocal, and transparent ways of ongoing engagement with Indigenous Peoples within the academy; and to pragmatically address claims of self-Indigenization” [18].

In efforts to establish mechanisms to collect disaggregated data, government entities and researchers need to acknowledge and understand the historical abuse of race-based data as a tool of oppression on racialized communities. Currently, there is no acknowledgement of the history of reproductive coercion that Indigenous and racialized womxn in North America have experienced and continue to experience. The current paradigm of SRHR research in understanding the lived experience (i.e., sociocultural, political, economic contexts) and effects of colonialism and discrimination are ignored when designing and conducting SHRH research. Therefore, it is critical for the government of Canada to commit to funding data collection efforts that enable researchers and policy makers to fully understand and communicate the impact of structural racism on SRHR outcomes among racialized communities. Re-iterating the importance of investing in scholars and community-based organizations from within these communities, to not only oversee the data collection processes, but also the use and access of this data, which in turn will enhance public accountability.

To conduct such ethically responsible research, scientists and community members must develop equal and cooperative partnerships. Utilizing the community's kinship network is important in garnering support for a study, recruiting study participants, and disseminating information. Whether it is unintended exclusion, intended exclusion or non-willingness to participate because of distrust of medical and research community by sub-groups, this is not a reason for excluding Black or Indigenous female participation in SHRH research. Willingness to participate by underrepresented groups is there if the study has relevance and cultural sensitivity to their own health and that of their community [19]. Primarily, SHRH research on Indigenous and Black womxn in Canada must recognize and respect their rights and acknowledge their perspectives regarding how the research work will be conducted in their communities.

Currently, any disaggregated data (i.e., census data and health surveys) are owned and stored by Statistics Canada. The government of Canada has enforced stringent policies surrounding access to this data [20], veiled in concern of privacy issues. However, there has been little to no evidence that this already encrypted data has ever been hacked. Indeed, population level data in the US and in other countries is fully accessible to the public. In Canada, however, unnecessary barriers prevent access for researchers, as only Canadian citizens are allowed access, requiring arduous applications, continuous approvals throughout the analysis stage as well as difficult accessibility (having to be in person).

## Conducting, mobilizing and translating knowledge for better health outcomes: the need for a reproductive justice framework in SRHR research in Canada

Knowledge translation and exchange as well as knowledge mobilization are methods in which research and knowledge are disseminated (i.e. transferred, translated, exchanged, co-produced) with the intention of practical application between researchers and users, including government, communities, policy makers, and professional entities [21, 22]. Hunter (2002) examined how racist epistemologies in sociological research impact the questions we ask, the theories we use, how we analyze the data, and the knowledge we disseminate and “the power relations we re/produce” [23]. Two issues arise in terms of research process and dissemination of findings for use. The first is regarding researchers in SRHR. To compete in the current research structures to ‘produce’ (i.e. receive grants, publish, achieve tenure), womxn scholars of colour have to work within existing colonial structures [24] where they face barriers to success through existing systemic racism, thus further marginalizing them, their work and their community. From this, stems the second issue, the SRHR research conducted. Without a reproductive justice lens in SRHR research, there is oversight in decision making, policies and programs that ignore IBPOC womxn’s lived experiences and health outcomes resulting from social injustices. The predominant research methods and subsequent dissemination of findings in SRHR are privileged and not representative of Indigenous, Black and racialized communities in Canada. Thus, decisions in healthcare policies and practices are being made based on research and evidence that does not consider the historical injustices and continues to sustain white privilege. Thus the need for a reproductive justice framework in SRHR research methodologies in Canada.

At the core of RJ is the link between reproductive rights and social justice [1]. This is the fundamental right to decide on one’s own reproductive health and not face the social, political and economic inequalities that make it difficult to access necessary or chosen sexual and reproductive health services [25]. There is a lack of research that incorporates an understanding of how race, class and gender inequalities constrain womxn’s control over their lives. Consequently, the negative SRHR health outcomes disproportionately affecting IBPOC womxn is perpetuated by the obstacles IBPOC womxn researchers face entering the research realm.

We continue to look for leading examples where this approach is making a difference in IBPOC womxn’s health in Canada. SRHR research with an RJ lens that considers lived experiences may be taking place across the country with promising results—we need this work to be funded and widely disseminated to tackle pervasive inequities. In the U.S., SisterSong and other reproductive justice groups are a strong representation of Indigenous womxn and womxn of colour who have worked for decades demanding consideration of how other factors (i.e., environment, poverty, employment, housing, immigration, sexual orientation, race, religion) affect reproductive health [26]. This network of organizations and individuals work together to “improve institutional policies and systems that impact the reproductive lives of marginalized communities”, recognizing the right and responsibility to represent their communities and put forth the SRH perspectives and needs of womxn of colour [27]. This political movement has worked hard for “access to specific community-based resources …[that include] high quality health care, housing, education, living wage, healthy environment and a safety net for times when these resources fail…(to provide] safe and dignified fertility management, childbirth and parenting.” [1]. An RJ perspective is critical for SRHR research in Canada to ensure a comprehensive and unrestricted access to reproductive healthcare for womxn of colour living in Canada.

COVID-19 has highlighted the disproportionate effect of the pandemic on Indigenous, Black, immigrant and refugee communities in Canada. More recently, we have seen a record number of pregnant womxn in intensive care units with the third wave [28]. As we continue on the same path that is compounded by lack of race-based data, the oversight to consider SRHR research through an RJ perspective by funders, health care providers, researchers and policy makers will only exacerbate the existing barriers and threats to IBPOC womxn’s health and safety.

## Indigenous and anti-racist methodologies: womxn of color in healthcare and the methodology of the privileged

A mindfulness to centralize race and gender in research projects, methodologies and theoretical approaches will open the space for inclusive approaches. A pioneer in Critical Race Theory, Kimberlé Crenshaw (1995) argued that empirical research should be re-centered to ideologies such as working-class cultures, feminist cultural research, the positionality of womxn of colour as well as race in social and cultural processes [29]. The inquiry considers the intersect of race, class and gender, and how it is expressed in our legal, health care and political systems. These social categories are *socially constructed*. Crenshaw (1991) began to research how law and legislation fail to consider factors such as how immigration status, language barriers, and power dynamics between men and womxn [30] affect outcomes in the supposedly neutral arena of legal affairs. It is this analysis that brings forward the question: *How does the empirical research in womxn’s sexual and reproductive health and rights policies access or produce research that is objective and representative of the needs of Indigenous, Black and womxn of colour?* What factors are at play in ethnocultural health differences? Discriminatory research methodologies in the study of SRHR affect the treatment and consideration of Indigenous, Black and racialized womxn.

These discriminatory methodologies contribute to the invisibility of womxn of color in the health care system by reducing their lived experiences to pathology. The current research methods can be viewed as creating knowledge and policies through *racial ventriloquism* [31] because current knowledge producing structures dominated by white researchers continue to lead and produce work on and for IBPOC communities. Privileging white research has, at times, justified systemic racism [32] and we continue to see this in the SRHR discourse, resulting in revictimization and continued racial trauma faced by Black and Indigenous womxn. In fact, the decolonial scholars have been inadequately self-critical of their own involvement with applying oppressive research methods [31]. The epidemiology of oppression is a contributing factor to the lack of research resources and accessibility for indigenous and womxn of colour. Crenshaw highlights structural and political intersectionality. The former focuses on the individual experience and intersections of multiple categories of differences faced by womxn of colour, while the latter focuses on how various social identity groups organize themselves between two or more political agendas or movements [30]. Subsequently, *these forms of intersectionality* lead to social structures and the parameters based on research that is discriminatory in manner, which lead to institutionalized racist practices or policies [24].

More recently, Harding (2015) eloquently discussed *standpoint theory* and the proposed *strong objectivity* advantages as an approach to conducting research [33]. The focus was on how “good science” as it currently stood held "sexist and adrocentric assumptions and practices that had shaped results of research.” (p. 2). Arising from gender-stereotyped lenses of disciplines that ignored and dismissed womxn’s conditions and lived experiences, feminists argued that societies were structured by inequalities and the current research and prevailing knowledge on which policies, programs and decisions were being made, represented the interest of the dominant groups [33]. Strong objectivity approach starts the research from outside the dominant white, Eurocentric conceptual framework to enable diverse critical perspectives and gain new insights.

Therefore, we are seeking to shift direction to a framework of *Reproductive Justice* [34, 35]. Moving towards a discourse of choice and a radical perspective that prioritizes the stories of womxn of color as the foundation for new knowledge [36]. RJ highlights the issues of social justice that are further linked to the knowledge being produced to address the negative reproductive health outcomes of Black, Indigenous and womxn of colour, while redirecting the attention to womxn’s health and reproductive rights from a decolonized perspective [1].

## Conclusion

Researchers and advocates in this field can no longer remain complacent in their assumptions and projections that research injustices do not exist in the SRHR field in Canada. A decolonizing framework for SRHR research is needed and should include a cultural and spiritual lens from inception of the study design, throughout planning and implementation, as well as during evaluation and knowledge dissemination efforts. Racialized womxn researchers must be the driving force behind this work to ensure relevant and meaningful outcomes for Canadian Indigenous, Black and racialized womxn's maternal and reproductive health and rights in Canada. In adopting this reproductive justice framework, there must be sufficient financial investment in research by and for Indigenous, Black and racialized voices. For this to materialize, we must address the lack of equitable processes, and hence allocations, within Canada’s health research funding matrices. If Canada adopts a reproductive justice framework—respecting the rights of individuals and their knowledge of their own SRHR—then funding Indigenous, Black and racialized researchers to conduct SHRH research in their own communities is paramount [37]. The research must encompass the resiliency of Black womxn and the Indigenous ways of knowing and being [2] and establishment of government funded initiatives that are focused on institutionalizing the dismantlement of racism in health [9].

## Data Availability

Not applicable.

## Footnotes

1 Disaggregated data: data that is broken down by demographics such as race, age, gender, and/or location.

2 The authors condemn the use of this colonist term, and its use within any legislative Acts. Any use of the term in this commentary refers to specific Acts and designations by the Canadian government.
